# Low Triiodothyronine Syndrome in Patients With Radiation Enteritis: Risk Factors and Clinical Outcomes an Observational Study

**DOI:** 10.1097/MD.0000000000002640

**Published:** 2016-02-12

**Authors:** Shengxian Fan, Xiaodong Ni, Jian Wang, Yongliang Zhang, Shen Tao, Mimi Chen, Yousheng Li, Jieshou Li

**Affiliations:** From the Department of Surgery, Jinling Hospital, Nanjing University School of Medicine, Nanjing, China.

## Abstract

The implications of low triiodothyronine syndrome (LT3S) in patients with radiation enteritis (RE) have not been properly investigated. As such, we conducted this cohort study to investigate the association between LT3S and RE, to explore the etiology of LT3S in RE, to evaluate the clinical features and clinical outcomes of LT3S patients, and to inspect the correlation of clinical variables and LT3S in RE.

This prospective study included 39 RE patients. Medical records and various laboratory parameters (including thyroidal, tumorous, nutritional, and radiotherapy variables) were collected in all participants.

Our results showed that the incidence of LT3S was 84.6% in patients with RE. Total protein (71.7 ± 5.7 vs 63.2 ± 9.6 g/L, *P* = 0.04) and albumin (ALB, 46.0 ± 4.6 vs 38.7 ± 5.3 g/L, *P* = 0.01) were significantly lower in LT3S group compared with those in euthyroid group. Standard thyroid-stimulating hormone index (−0.89 ± 2.11 vs −2.39 ± 1.33, *P* = 0.03) and sum activity of deiodinases (19.74 ± 4.19 vs 12.55 ± 4.32 nmol/L, *P* = 0.01) were significantly lower in LT3S group. Patients with LT3S suffered longer duration of hospitalization (48.25 ± 23.29 days in LT3S vs 26.75 ± 10.56 days in euthyroid, *P* = 0.036). Low serum ALB (β = 0.694, 95% CI = 0.007–0.190, *P* = 0.037) was the only significant predictor of LT3S.

LT3S was common in RE patients. A hypodeiodination condition and a potential pituitary-thyrotroph dysfunction might play a role in the pathophysiology of LT3S in RE. Worse nutritional status and clinical outcomes were confirmed in RE patients with LT3S. Furthermore, total protein and ALB were observed as protective and differentiating parameters of LT3S in RE. In summary, this was the 1st investigation to evaluate the clinical correlation between RE and LT3S, investigate the prevalence of LT3S in RE, and explore the pathogenesis of LT3S, despite the limitation of a relatively small sample size. These results will hopefully encourage future research to place greater emphasis on early identification of LT3S in RE patients.

## INTRODUCTION

Low triiodothyronine syndrome (LT3S), a condition characterized by a decrease in serum triiodothyronine (T3) level with a normal or slightly low level of thyroxine (T4) and thyroid-stimulating hormone (TSH) in the absence of any underlying intrinsic thyroid disease, is variously known as the “nonthyroidal illness syndrome,” or the “euthyroid sick syndrome.”^1^ This condition is frequently detected in various acute and chronic illnesses, such as sepsis,^2^ myocardial infarction,^3^ and starvation.^4^ Reichlin et al^5^ first noticed that some aspects of thyroid hormone metabolism might change during many chronic illnesses as early as 1973. Data have shown that about 35%–70% of acute and chronic critical conditions were associated with a significant abnormal thyroid hormone metabolism, of which LT3S was the most common type.^3^ The underlying mechanism of this disorder was explained by multifactorial causes including modifications to the hypothalamic-pituitary axis, altered binding of thyroid hormone to circulating binding proteins, modified entry of thyroid hormone into tissue, changes in thyroid hormone metabolism due to modified expression of the intracellular iodothyronine deiodinases, and changes in thyroid hormone receptor expression or function.^6^ In addition, these changes have been shown to be associated with disease severity and have been connected with poor short-term prognosis.^7,8^

Radiation enteritis (RE) is by simple definition an inflammatory process occurring at the level of the intestines as a response to abdominal or pelvic radiation energy exposure.^9^ Due to the sensitivity of organs to radiation, volume of irradiated tissue, and some patient characteristics, RE can present as either an acute or a chronic syndrome. The acute form presents within hours to days of radiation exposure and typically resolves within few weeks.^10^ However, the chronic form may present as early as 2 months or as long as 30 years after exposure.^11,12^ The mechanisms involved in the initiation and progression of radiation-induced intestinal injury remain largely unexplored; however, extensive data suggest that RE may arise as a result of the interaction of chronic inflammation.^13^ Moreover, certain mucosal cytokines such as interleukin-1 (IL-1), interleukin-6 (IL-6), and tumor necrosis factor-α are significantly elevated in RE patients.^14^ In RE, mucosal atrophy and dense infiltration with polymorphonuclear leukocytes occurs. As a result of stem cell loss and reduction in crypt mitoses, epithelial denudation and mucosal ulceration occurs. The resultant epithelial dysfunction leads to nutrient and fluid loss, whereas the associated increase in intestinal permeability to intestinal pathogens can exacerbate mucosal inflammation.^15^ Recent experimental studies have indicated that LT3S is associated with inflammation and nutritional status.^3,16^ What is more, previous studies have recognized the association of LT3S and chronic inflammatory diseases, such as inflammatory bowel disease,^17,18^ and raised the possibility that inflammatory cytokines may be predisposing factors for LT3S.^19^ Based on these aspects, we hypothesize that LT3S might be associated with RE.

However, there was still no systematic analysis of LT3S in patients with RE, the incidence, the underlying mechanisms, the clinical outcomes, and clinical variables of LT3S in RE have not been completely established. To address this existing gap in knowledge, we investigated the association between LT3S and RE, and the clinical characteristics of RE patients with LT3S compared with euthyroid RE patients.

## MATERIALS AND METHODS

### Study Design

This was a prospective single-center study, designed according to the ethical principles outlined by the Declaration of Helsinki and approved by the local ethics committee of Jinling Hospital, to evaluate the incidence, the underlying mechanisms, the clinical outcomes, and clinical variables of LT3S in RE patients. All the participants provided written informed consent.

### Participants

We prospectively collected 39 patients who were definitely diagnosed as RE at our center between July 2014 and July 2015. The diagnosis of RE was established in accordance with clinical manifestations, radiologic, endoscopic, and histopathologic evidences.

The exclusion criteria primarily consisted of the following aspects: previous disease of thyroid, pituitary, or hypothalamus; previous disease of rheumatoid arthritis, systemic lupus erythematosus, and inflammatory bowel disease; tumor recurrence; craniocerebral injury; gestational or lactational period; a medication history of thyroidal hormone or antithyroid drugs in the past month; an attack of coronary heart disease, myocardial, or cerebral infarction in the past month; an intracranial infection or hemorrhage in the past month; and radiotherapy of head and neck malignancies.

### Data Collection and Laboratory Parameters

All enrolled patients underwent anamnesis and physical examination, anthropometric measurement, and biochemical screening. Demographic and clinical data such as age and sex, medical records, body mass index (BMI), and Acute Physiology and Chronic Health Evaluation II (APACHE II) score were collected on admission. Clinical outcomes, such as presentation of diarrhea and obstruction, duration of hospital stays, and probabilities of operation were evaluated on admission and after discharge. Fasting blood samples were obtained in the morning following admission. Blood was rapidly centrifuged and serum was frozen at −20 °C. The samples from all individuals for each parameter were analyzed in a single batch. Laboratory tests, including hemoglobin (Hb), total protein (TP), albumin (ALB), white blood cell (WBC) counts, and C-reactive protein (CRP), were performed within 2 hours using Bayer ADVIA Centaur automated immunoassay system (Bayer, Leverkusen, Germany). Tumor markers, computed tomography (CT) scan, and positron emission tomography-computed tomography (PET-CT) scan were measured and performed during the first 24 hours of hospitalization. Serum concentrations of free triiodothyronine (FT3), total triiodothyronine (TT3), free thyroxin (FT4), total thyroxin (TT4), TSH, antithyroperoxidase antibody (anti-TPO Ab), antithyroglobulin antibody (anti-TG Ab), antithyroid-stimulating hormone receptor antibody (anti-TR Ab), and antithyromicrosome antibody (anti-TM Ab) were measured via chemiluminescentmicroparticle immunoassay (Amersham Pharmacia Biotech, Aylesbury, UK). Reference limits were as follows: T3: 1.23 to 3.07 nmol/L; T4: 0.071 to 0.161 μmol/L; FT3: 3.8 to 6.5 pmol/L; FT4: 7.9 to 17.2 pmol/L; TSH: 0.3 to 4.5 mIU/L; anti-TPO Ab: <30 IU/mL; anti-TG Ab: 0.2 to 30 IU/mL; anti-TR Ab: 0.11 to 30 IU/mL; and anti-TM Ab: 0.2 to 10 IU/mL.

To investigate the underlying etiology of LT3S in RE, we evaluated the following variables of thyroid metabolism: standard TSH index (sTSHI) as an evaluation of central component of LT3S, especially markers of pituitary-thyrotroph function; sum activity of deiodinases (GD) as a variable for deiodination function; thyroid's secretory capacity (GT) as an evaluation of thyroid secretory status; and the ratios of TT3/FT3 and TT4/FT4 as evaluations of protein binding of thyroid hormones. The sTSHI was defined as sTSHI = (TSH-2.70)/0.676 (reference range: −2 to +2).^20^ Additionally, the parameters of GD and GT were calculated as GD = [β_31_ × (K_M1_ + FT4) × TT3]/(α_31_ × FT4) (reference range: 20–40 nmol/s) and GT = [β_T_ × (D_T_ + TSH) × TT4]/(α_T_ × TSH) (reference range: 1.41–8.67 pmol/s), respectively. Constants in the equations were as follows: β_T_ = 1.1 × 10^–6^/s, D_T_ = 2.75 mU/L, α_T_ = 0.1/L, β_31_ = 8 × 10^–6^/s, K_M1_ = 5 × 10^–7^ mol/L, and α_31_ = 0.026/L.^21^

### Statistical Analysis

All continuous results were presented as mean ± SEM. Differences between groups were tested by Student's *t*-test. Frequencies were analyzed by Chi-square test. Univariate and multivariate linear regression analysis was performed to investigate the risk factors of LT3S in RE, and the correlation between laboratory parameters and clinical variables. The statistical analysis was performed with the SPSS statistical software (version 20; SPSS, Chicago, IL). Statistical significance was accepted at the *P* < 0.05 level.

## RESULTS

**FIGURE 1:**
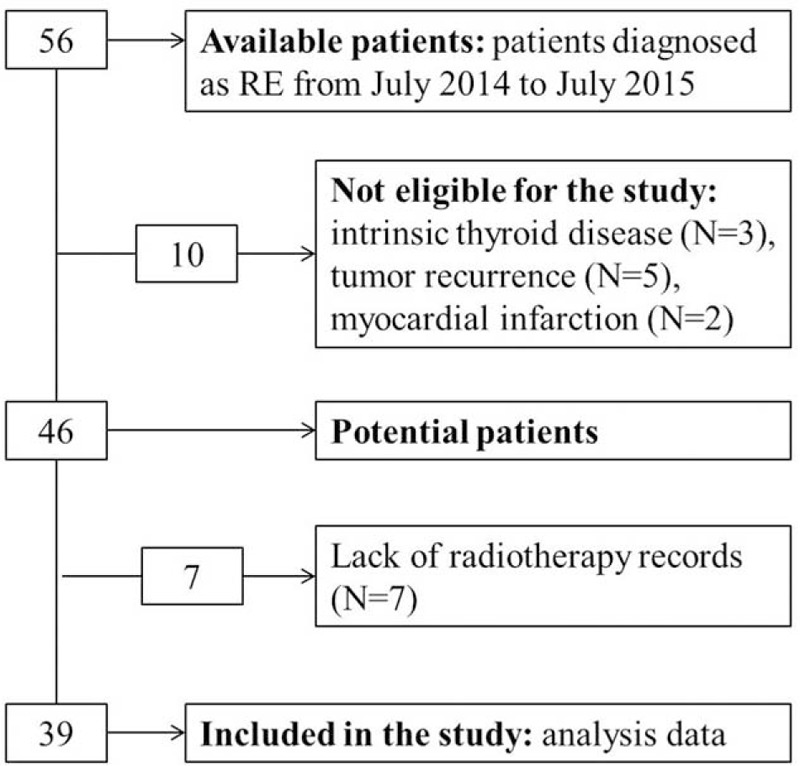
Flowchart: screening of subjects for data analysis. RE = radiation enteritis.

From July 2014 to July 2015, 56 RE patients had been admitted to department of general surgery, Jinling Hospital. Patients excluded from this study were those with a history of thyroid diseases (n = 3), tumor recurrence (n = 5), a history of myocardial infarction (n = 2), and lack of radiotherapy records (n = 7). The remaining 39 patients were included in this study (Figure 1). Among the 39 RE patients, 24 were primary cervical cancer, 8 were primary rectal cancer, 2 were primary colon cancer, 1 was primary prostate cancer, and 4 were other malignancies. The mean age was 53.9 years old (range 32–76 years), 79.5% of patients were female. Table 1 detailed demographic and clinical characteristics of 39 patients enrolled in this study.

**TABLE 1:**
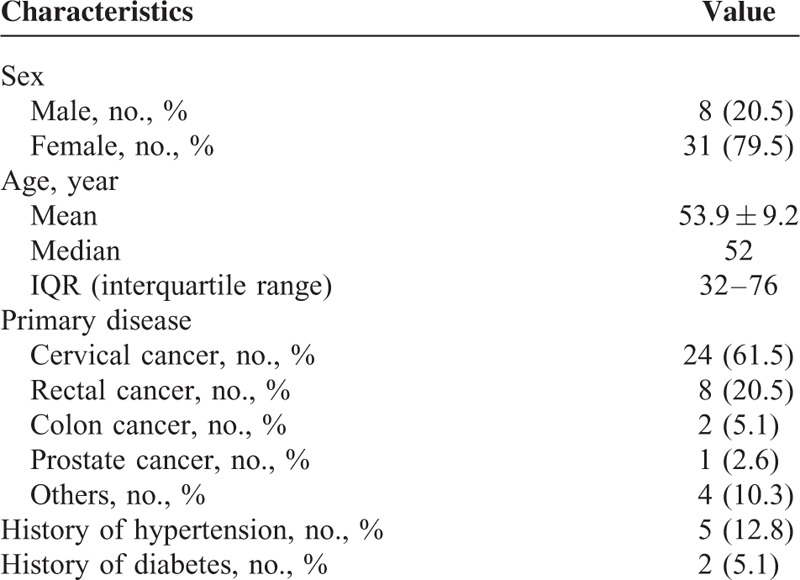
Clinical Characteristics of Study Population

The serum levels of anti-TG Ab, anti-TPO Ab, anti-TM Ab, and anti-TR Ab were measured in all 39 RE patients. Mean ± SEM were 6.77 ± 8.70, 8.60 ± 6.05, 3.81 ± 2.71, and 16.77 ± 1.63 IU/mL, respectively. Serum levels of anti-TG Ab, anti-TPO Ab, anti-TM Ab, and anti-TR Ab were normal in each of the 39 individuals.

**FIGURE 2:**
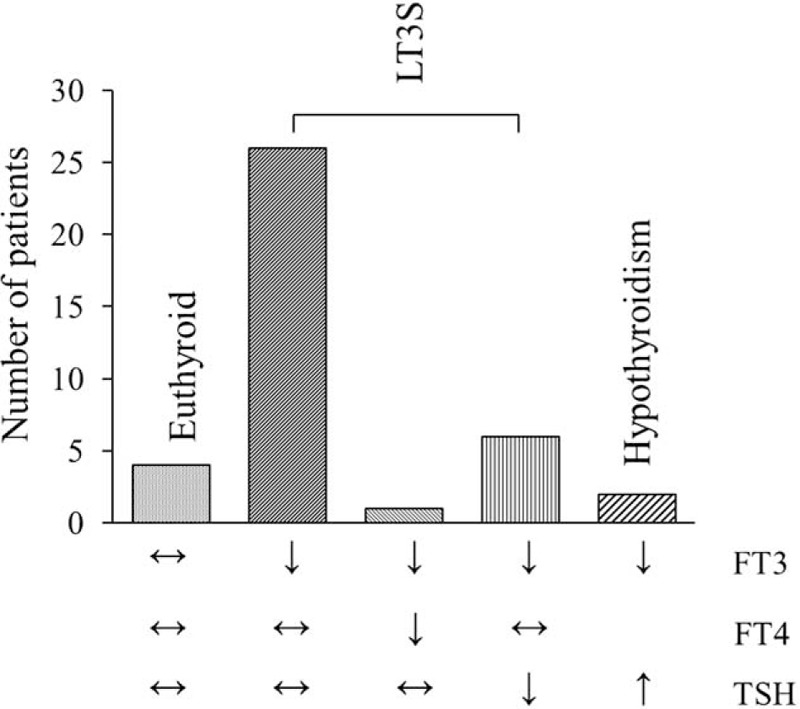
The distribution of thyroidal laboratory values in enrolled patients. FT3 = free triiodothyronine, FT4 = free thyroxin, LT3S = low triiodothyronine syndrome, TSH = thyroid-stimulating hormone.

Of the enrolled 39 patients, 4 (10.3%) patients were euthyroid with normal FT3, FT4, and TSH levels. Thirty-three (84.6%) patients were diagnosed with LT3S, which was defined as a low serum FT3 level without an elevated TSH level. Among them, 1 patient was with concomitant low FT4 level, and 6 patients were with concomitant low TSH level. Two (5.1%) patients were diagnosed with hypothyroidism because of a low FT3 and increased TSH level (Figure 2).

We further compared the differences between euthyroid and LT3S groups in RE patients. Table 2 showed the demographics and clinical variables between these 2 groups. Age, sex distribution, BMI, APACHEII score, history of hypertension and diabetes, radiotherapy parameters, Hb, WBC, and CRP were similar between euthyroid and LT3S patients. In contrast, TP (71.7 ± 5.7 vs 63.2 ± 9.6 g/L, *P* = 0.04) and ALB (46.0 ± 4.6 vs 38.7 ± 5.3 g/L, *P* = 0.01) were significantly lower in LT3S group compared with those in euthyroid group, suggesting a worse nutritional condition in patients with LT3S. Meanwhile, FT3 (4.63 ± 0.73 vs 3.01 ± 0.61 pmol/L, *P* = 0.00), TT3 (1.64 ± 0.14 vs 0.96 ± 0.30 nmol/L, *P* = 0.00), TSH (2.10 ± 1.43 vs 1.08 ± 0.90 mIU/L, *P* = 0.03), sTSHI (−0.89 ± 2.11 vs −2.39 ± 1.33, *P* = 0.03), and GD (19.74 ± 4.19 vs 12.55 ± 4.32 nmol/L, *P* = 0.01) were significantly lower in LT3S group, whereas other thyroid hemostasis parameters were similar between these 2 groups (Table 2). The decreased levels of sTSHI (−2.39) and GD (12.55 nmol/L) presumed a downregulation of pituitary-thyrotroph function and hypodeiodination in LT3S group, which meant a hypodeiodination condition and a potential pituitary-thyrotroph dysfunction may play a role in the pathophysiology of LT3S in RE.

**TABLE 2:**
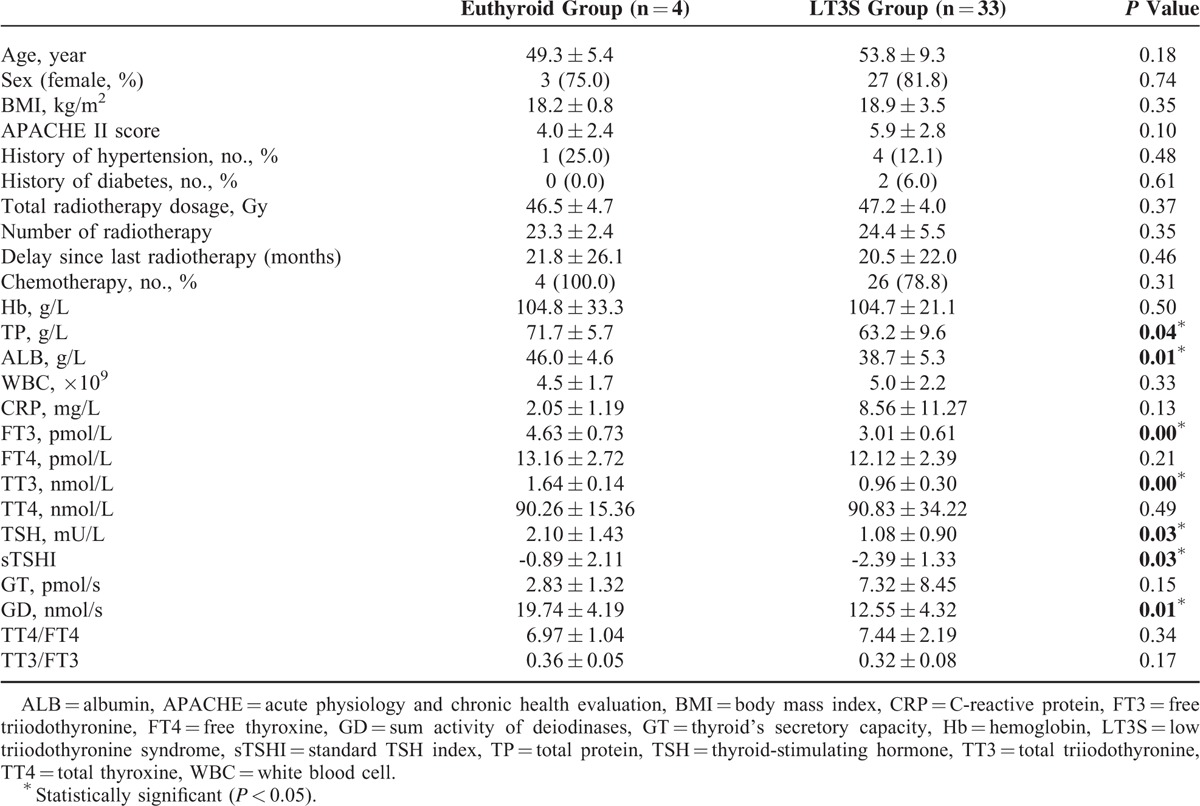
Demographics and Clinical Variables Between Euthyroid and LT3S Groups

To further investigate the correlation between clinical variables and thyroid parameters, we performed univariate and multivariate linear regression analysis. Clinical variables included age, sex distribution, BMI, APACHEII score, WBC counts, Hb, CRP, TP, ALB, and radiotherapy parameters. TP (β = 0.331, 95% CI = 0.001–0.055, *P* = 0.046) and ALB (β = 0.441, 95% CI = 0.019–0.106, *P* = 0.006) emerged as potential predictors, which meant both TP and ALB were with a close relationship to serum concentration of FT3. Then we performed a multiple linear regression model to further investigate factors that were actually associated with FT3 level. In multivariate analysis, low serum ALB concentration (β = 0.694, 95% CI = 0.007–0.190, *P* = 0.037) was the only significant predictor of LT3S (Table 3).

**TABLE 3:**
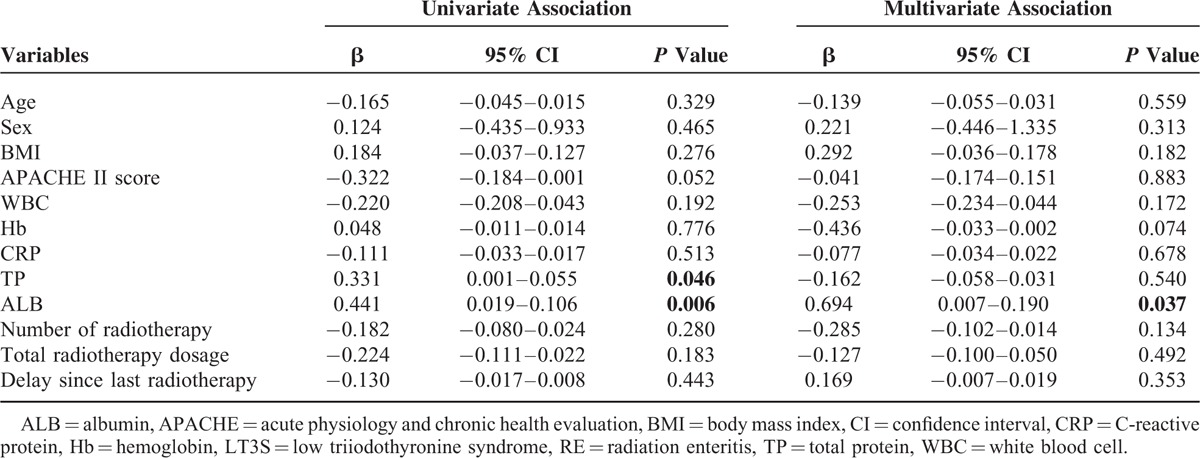
Association of Clinical Variables With LT3S in RE

Clinical outcomes, including presentation of diarrhea and obstruction, days of hospitalization, and percentages of operation were further analyzed. As shown in Table 4, the patients with LT3S displayed a statistically longer stay of hospitalization compared with that in euthyroid group (48.25 ± 23.29 days in LT3S vs 26.75 ± 10.56 days in euthyroid, *P* = 0.036). Differences of presentation of diarrhea (81.8% in LT3S vs 50.0% in euthyroid, *P* = 0.923) and obstruction (60.6% in LT3S vs 50.0% in euthyroid, *P* = 0.683), and percentages of operation (78.8% in LT3S vs 50.0% in euthyroid, *P* = 0.205) between LT3S and euthyroid groups were not confirmed (Table 4). However, even the differences were not significant enough to reach statistical standard, the present study still implied a more severe presentation of diarrhea (diarrhea >5 times a day, 15.2% in LT3S vs 0.0% in euthyroid, respectively), greater percentages of obstruction, and higher probabilities of operation in LT3S individuals.

**TABLE 4:**
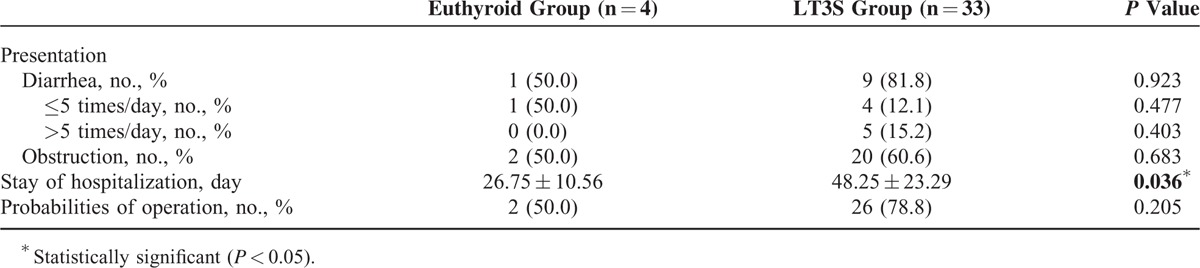
Clinical Outcomes Between Euthyroid and Low Triiodothyronine Syndrome (LT3S) Groups

## DISCUSSION

This was the first investigation to evaluate the clinical correlation between RE and LT3S, investigate the prevalence of LT3S in RE, and explore the pathogenesis of LT3S. In the present study, we prospectively collected 39 patients in our center. We demonstrated that the incidence of LT3S in RE was 84.6%, which was observed higher than the incidence of other thyroid disorders, including hypothyroidism (5.1%). Of the 33 LT3S patients, 6 patients were with concomitant low TSH concentrations, thereby indicating changes in the hypothalamic-pituitary regulation; 1 patient was with concomitant low FT4 concentration, which may also be due to an impaired hypothalamic or pituitary regulation.^22^ Despite these changes, serum concentrations of anti-TG Ab, anti-TPO Ab, anti-TM Ab, and anti-TR Ab were normal in all patients enrolled in our study, which conformed there was no autoimmune thyroid disease in all the 39 individuals.

In several illnesses, LT3S has been described as decreased T3 with a normal or low concentration of T4 and a normal or low concentration of TSH. Previous studies reported that about 70% of patients hospitalized with various illnesses were affected by LT3S.^23^ Although this pattern has been clinically described for more than 30 years, it still remains controversial what is the likely mechanism behind these changes and whether the presence of LT3S belongs to a result of a maladaptive or a protective process.^24,25^ Multiple factors such as dysfunction of hypothalamic-pituitary-thyroid axis and altered peripheral thyroid hormone metabolism are demonstrated to be essential mechanisms in the pathogenesis of LT3S.^26^ To the best of our knowledge, there is no study reporting occurrence rate of LT3S in RE in literature and there is a lack of study focusing on the correlation of LT3S and RE. However, our study displayed LT3S occurred frequently with a prevalence of (84.6%) in RE, which was higher than previously reported in hospitalized cardiac patients (30%) and in acutely ill elderly patients (32%),^27,28^ reminding clinical physicians to be aware of the possibility of LT3S occurrence in RE.

In this study, the age and sex distribution, the BMI and APACHE II score, the presence of diabetes and hypertension, and the radiotherapy parameters had no influence on serum concentrations of T3, T4, and TSH. Low serum concentrations of TP and ALB were recognized as risk factors for LT3S in RE in current study, suggesting a worse nutritional status in patients with LT3S. Patients with high serum concentrations of TP and ALB were less likely to have LT3S. This emphasizes the critical importance of routine surveillance of serum TP and ALB concentrations in RE patients, which may be helpful for clinical physicians to identify the occurrence of LT3S in RE. Of note, we did not evaluate the efficacy of ALB supplementation for improving clinical variables and prognosis, and the effect of ALB supplementation on thyroid hormone metabolism in RE patients with LT3S. Larger prospective, multicenter studies were expected to explore these issues.

Furthermore, we tried to explore the pathogenesis of LT3S in RE. LT3S was generally characterized by 3 components, which could occur solely or in combination:

1. Impaired protein binding of thyroid hormones, thus the ratios of TT3/FT3 and TT4/FT4 were measured in our study cohort to reveal the binding condition
2. Central hypothyroidism (transient thyrotropic insufficiency), accordingly parameters of sTSHI and TSHI were measured to assess the pituitary thyrothroph function
3. Reduced formation of T3 with concomitantly increased conversion of rT3, thus the parameter of GD was calculated to assess the deiodination status^21^

In addition, we calculated the parameter of GT to evaluate the thyroid function. Our results demonstrated a reduced GD level in LT3S group compared with euthyroid group. Combining with the consideration of the decreased concentration of FT3, we speculated that a decreased deiodination condition may be partially responsible for the occurrence of LT3S in patients with RE. In addition, another key finding in this study was a lower sTSHI in LT3S group, suggesting a potential pituitary-thyrotroph dysfunction may also play a role in the pathogenesis of LT3S in RE patients. However, since the parameters of TT3/FT3 and TT4/FT4 between these 2 groups were not significant enough to reach statistical standard, larger multicenter studies were expected to further explore the pathogenesis of LT3S in RE.

Meanwhile, our results showed that patients in the LT3S group displayed longer stays in hospital compared with the euthyroid group. Patients in our cohort with LT3S also had a more severe presentation of diarrhea, greater percentages of obstruction, and higher probabilities of operation than those with normal thyroid function, although the differences were not statistically significant. We speculated that the limited sample size of the present study might be the primary factor in the prevention of discovery, and believed that larger multicenter studies would be a great advance for exploring the clinical variables and prognosis between euthyroid and LT3S groups. Our results were consistent with previous published studies of critical and chronic illnesses.^8,27,28^

Treatment of patients affected by LT3S with thyroid hormones has been controversially discussed, and there were only a few studies in humans with small numbers available so far.^29–32^ Treatment with T3 has been investigated in coronary artery bypass surgery^31,32^ and in burn patients,^30^ but its clinical benefit has not been repeatedly shown yet. For example, in patients undergoing heart surgery^32,33^ or in patients with heart failure,^34^ thyroid hormone replacement therapy was safe and improved hemodynamic function; however, the benefit with respect to mortality remains less clear. Others have reported that thyroid hormone replacement therapy did not improve clinical outcomes in patients with burn injuries^30^ and in intensive care unit patients.^29^ Another possible treatment includes the substitution of T4, which restored serum T4 concentrations but reduced TSH concentrations and failed to show a clinically beneficial effect.^29^

We are aware that some limitations of this study merit consideration. First, as a single-center study, a potential selection bias might limit the extrapolation of our results. However, all measurements were performed within 24 hours of admission, which makes our study homogeneous. Second, as only 39 RE patients were enrolled in our study, the sample size might be a little small to detect the real diversity of duration of hospitalization and possibilities of operation between LT3S and euthyroid groups. In addition, we did not have the opportunity to measure levels of proinflammatory cytokines, which might have given slightly different results of our study. Also, whether replacing thyroid hormones and raising FT3 into the normal range could help to improve the outcomes of RE patients in LT3S group is unknown.

## CONCLUSIONS

In summary, LT3S is a common complication in RE patients and is independently associated with increased risk for worse nutritional status and clinical outcomes. The present findings also indicate that a potential pituitary-thyrotroph dysfunction and a hypodeiodination condition may contribute to the pathogenesis of LT3S in RE. Furthermore, TP and ALB are recognized as protective and distinguishing factors of LT3S in RE. However, due to the limitations of this study, further study will be needed to clarify whether LT3S in RE is a useful compensatory mechanism to counteract excessive catabolism or an unfavorable adaptation, and whether it is necessary to treat LT3S with hormone replacement therapy in RE patients.
